# Recent advances in genome mining of secondary metabolites in *Aspergillus terreus*

**DOI:** 10.3389/fmicb.2014.00717

**Published:** 2014-12-23

**Authors:** Chun-Jun Guo, Clay C. C. Wang

**Affiliations:** ^1^Department of Pharmacology and Pharmaceutical Sciences, School of Pharmacy, University of Southern CaliforniaLos Angeles, CA, USA; ^2^Department of Chemistry, College of Letters, Arts, and Sciences, University of Southern CaliforniaLos Angeles, CA, USA

**Keywords:** genome mining, *Aspergillus terreus*, secondary metabolites, natural products

## Abstract

Filamentous fungi are rich resources of secondary metabolites (SMs) with a variety of interesting biological activities. Recent advances in genome sequencing and techniques in genetic manipulation have enabled researchers to study the biosynthetic genes of these SMs. *Aspergillus terreus* is the well-known producer of lovastatin, a cholesterol-lowering drug. This fungus also produces other SMs, including acetylaranotin, butyrolactones, and territram, with interesting bioactivities. This review will cover recent progress in genome mining of SMs identified in this fungus. The identification and characterization of the gene cluster for these SMs, as well as the proposed biosynthetic pathways, will be discussed in depth.

## Introduction

Filamentous fungi, such as species found within the genus *Aspergillus*, are known to produce a wide variety of natural products displaying a broad spectrum of biological activities. Genome sequencing of members in the genus *Aspergillus* revealed that the number of secondary metabolite (SM) genes or gene clusters greatly exceeds the number of SMs identified so far. This suggests that more types of SMs still remain to be discovered. Efficient and high-throughput genome sequencing techniques have now greatly facilitated the genome mining of SMs identified in *Aspergillus* species. More and more studies have shown that most fungal SM genes are clustered, often separated from each other by less than 2 kb within the chromosome (Keller et al., 2005). Striking examples of fungal SM clusters include those responsible for the biosynthesis of fumonisin (Proctor et al., 2003) and sterigmatocystin (Brown et al., 1996). The clustering of these SM genes is fortuitous for researchers since upon discovering one responsible gene within a cluster, other genes that are within close proximity of the identified genes may be involved in the biosynthesis of the same natural product.

Analysis of the fungal genome makes it feasible to characterize these SM gene clusters via genetic manipulations. Verification of function of the relevant genes can be achieved via several approaches. These approaches include targeted deletion or over-expression of the relevant genes in the native organism, heterologous expression in an alternative host, or *in vitro* biochemical characterization. Recent advances in the genome editing of *Aspergillus nidulans* have greatly expedited the SM genome mining of this fungal species (Sanchez et al., 2012). One advance is the development of a fusion PCR technique that allows quick synthesis of linear PCR fragments that are used in the transformation of filamentous fungi (Yu et al., 2004; Szewczyk et al., 2006). Another advance concerns the establishment of an efficient gene targeting system in the fungus *A. nidulans*. This is accomplished via targeted deletion of the *A. nidulans* homolog (*nkuA*) of the human KU70 gene which is essential for non-homologous end joining of DNA in DNA double-strand breaks (Nayak et al., 2006; Szewczyk et al., 2006). Later, this *ku70* deletion toolbox was expanded to other *Aspergillus* species and boosted the gene targeting efficiency in these species (Kuck and Hoff, 2010).

The fungus *Aspergillus terreus* is known to produce lovastatin, which became the first cholesterol-lowering drug of its class approved for human use in the United States. Besides lovastatin, *A. terreus* produces a number of biologically relevant compounds such as sulochrin, terretonin, asterriquinone, and butyrolactone. The strain *A. terreus* NIH 2624, a patient isolate, was sequenced by the Broad Institute as part of the Broad Fungal Genome Initiative. A 10 × coverage genome sequence has been completed, and the data are publicly available through the Broad Institute website. Analysis by Secondary Metabolite Unique Regions Finder (SMURF) showed that *A. terreus* NIH2624 contains 28 polyketide synthase (PKS) genes, 22 non-ribosomal peptide synthetase (NRPS) genes, one hybrid PKS/NRPS gene, two PKS-like genes, and 15 NRPS-like genes (Khaldi et al., 2010). This review will cover the most current knowledge of the genome mining of SMs produced by *A. terreus*. The review consists of three major sections: genome mining of PKS-derived natural products, genome mining of NRPS-derived natural products, and genome mining of hybrid PKS-NRPS derived natural products by *A. terreus*.

## Genome mining of PKS-derived natural products in *A. terreus*

### Biosynthesis of lovastatin in *A. terreus*

**Figure 1:**
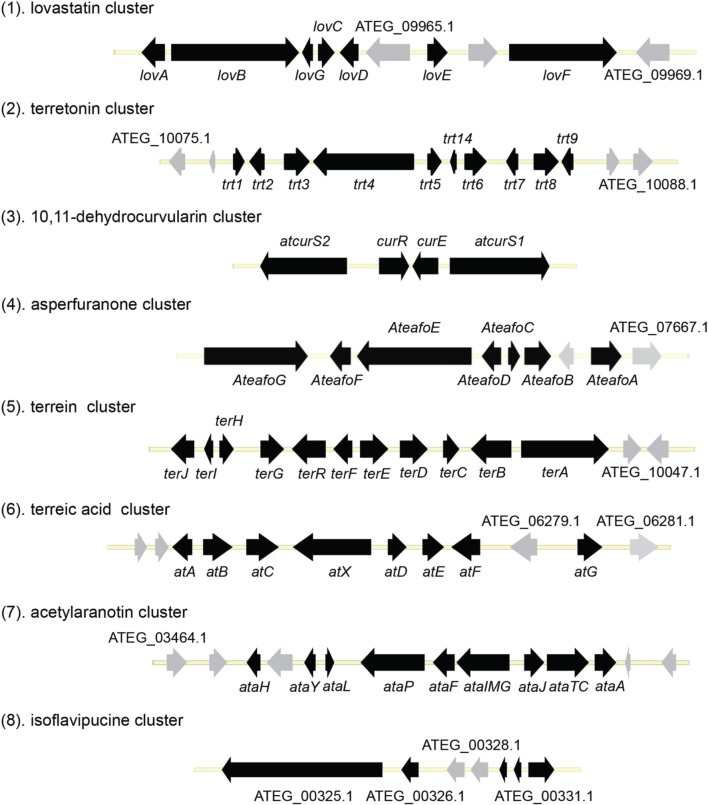
**Organization of the secondary metabolite gene clusters identified in *A. terreus***.

**Figure 2:**
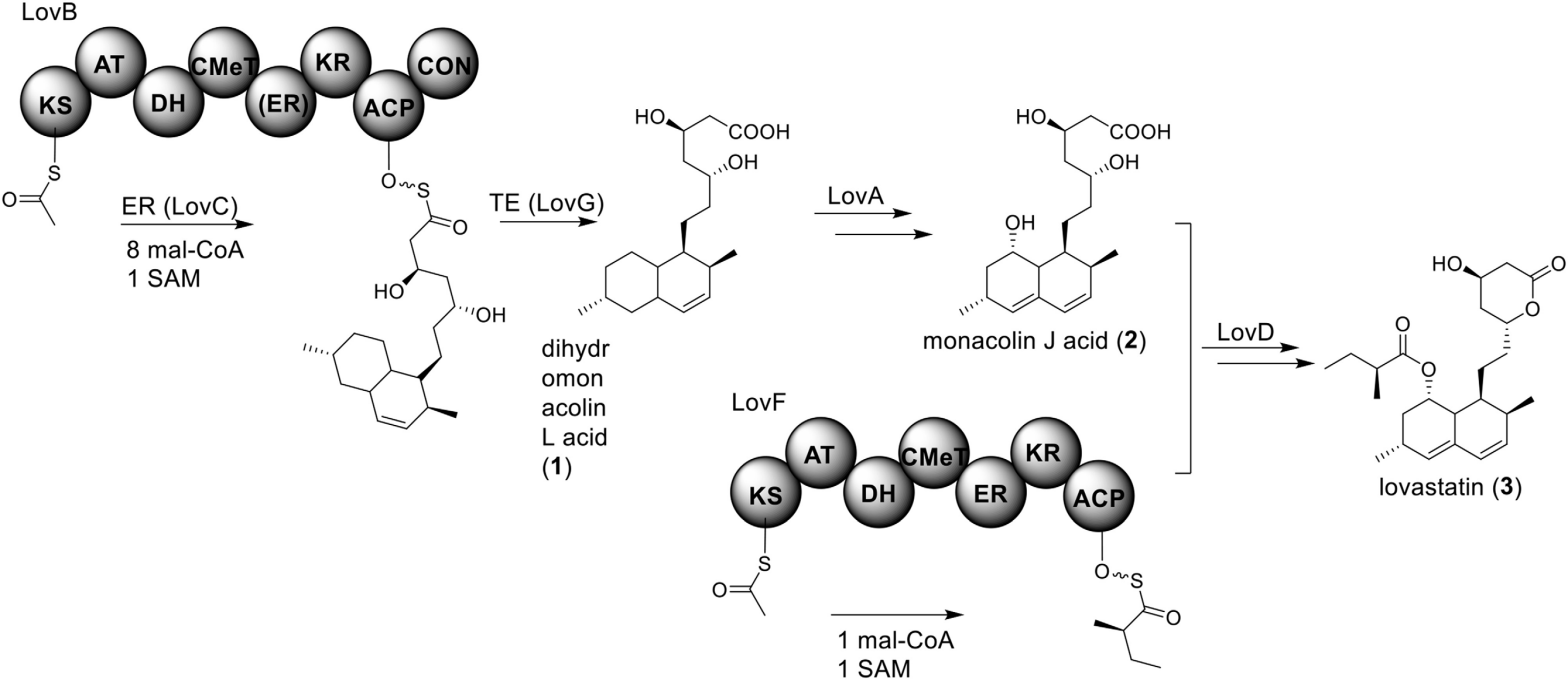
**Biosynthesis of lovastain in *A. terreus***.

Perhaps the most well-known molecule produced by *A. terreus* is lovastatin. Lovastatin is an inhibitor of the enzyme (3*S*)-hydroxymethylglutaryl-coenzyme A (HMG-CoA) reductase which reduces HMG-CoA to mevalonate. The biosynthesis of lovastain has been extensively reviewed in literature reports (Hill, 2006; Cox, 2007; Campbell and Vederas, 2010; Chiang et al., 2010; Chooi and Tang, 2012). Two highly reducing PKSs (HR-PKSs), LovB (lovastatin nonaketide synthase) and LovF, play critical roles in the formation of the lovastatin core structure (Figure 1). In the pathway, the HR-PKS LovB, together with the trans-acting enoyl reductase (ER) protein LovC, are responsible for the production of dihydromonacolin L acid (**1**). The release of **1** from LovB is catalyzed by a thioesterase LovG (Xu et al., 2013a). The oxidative conversion of **1** to monacolin J acid (**2**) involves LovA. The other HR-PKS LovF synthesizes the 2-methylbutyrate moiety of lovastatin (**3**) and the covalent attachment of this moiety to **2** is catalyzed by a transesterase, LovD (Figure 2). Recent efforts in engineering LovD using a directed evolution strategy generated a mutant LovD containing 29 point mutations that greatly improves (~1000 fold) the protein's efficiency in the synthesis of the drug simvastatin (Jimenez-Oses et al., 2014), representing an excellent example of how the combination of synthetic biology and protein chemistry may facilitate drug discovery and production.

### Biosynthesis of terretonin in *A. terreus*

**Figure 3:**
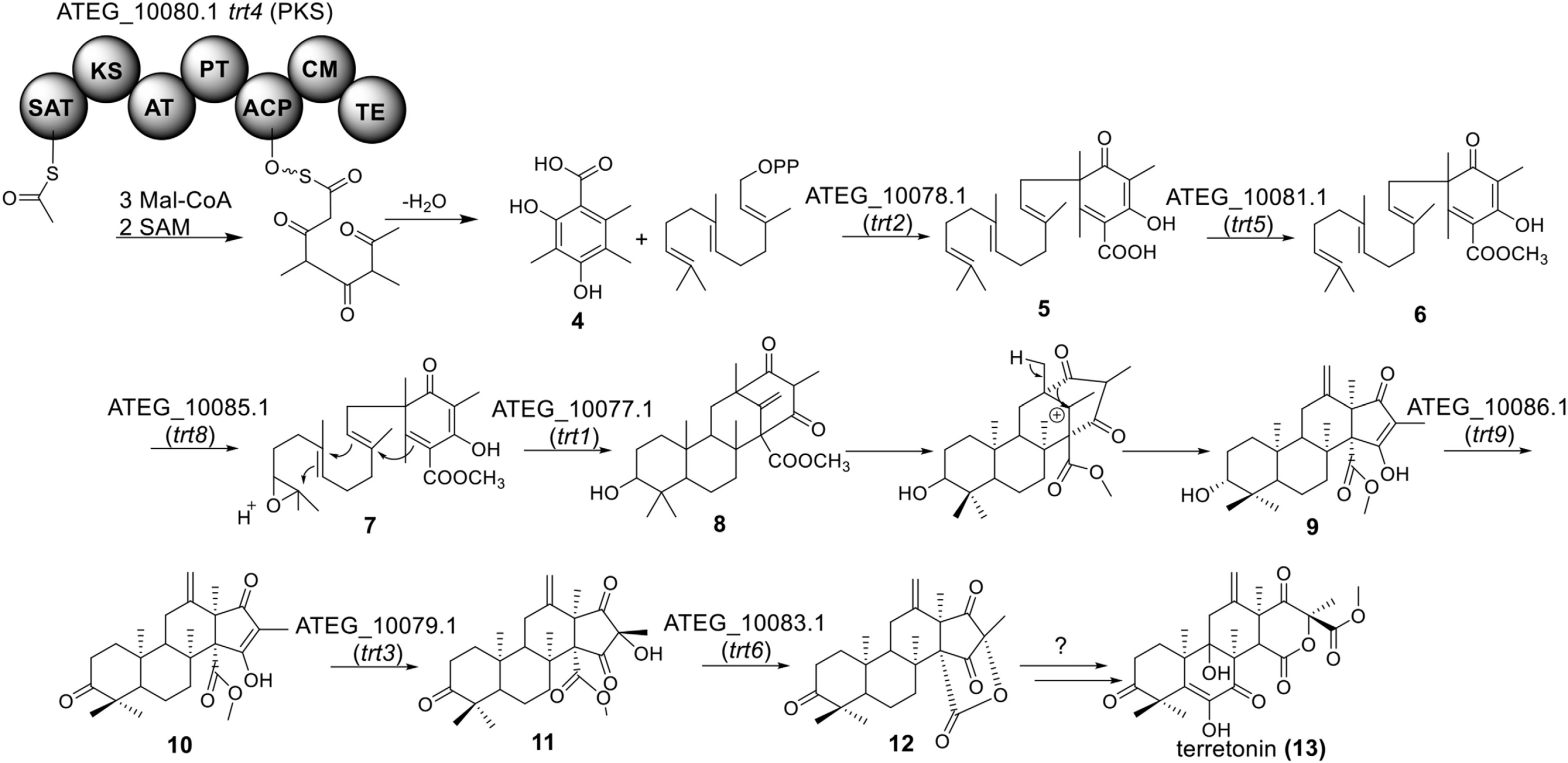
**Biosynthesis of terretonin in *A. terreus***.

Terretonin is one type of meroterpenoids with mixed origins (Figure 3). Previous studies by Simpson and Vederas in the 1980's using isotopic labeled precursors have shown that terretonin originates from both polyketide and terpenoid pathways (McIntyre et al., 1982, 1989). Recent genome mining studies revealed that the terretonin cluster contains a total of 10 genes (Guo et al., 2012). The cluster includes a non-reducing PKS (NR-PKS) gene, *trt4* (ATEG_10080.1), and a prenyltransferase gene, *trt2* (Figure 1). Trt4 is responsible for the biosynthesis of 3,5-dimethylorsellinic acid (**4**) and Trt2 catalyzes the alkylation of compound **4** to give **5** (Figures 1, 3) (Guo et al., 2012; Itoh et al., 2012). The following methylation (**5** to **6**), epoxidation (**6** to **7**) and cyclization (**7** to **8**) are proposed to be catalyzed by proteins encoded by *trt5*, *trt8*, and *trt1*, respectively (Guo et al., 2012; Matsuda et al., 2012). Interestingly, the Abe group discovered that the methylation step catalyzed by Trt5 is an essential step for cyclization (Matsuda et al., 2012). Upon formation of the tetracyclic precursor, Trt9 is proposed to oxidize compound **9** to **10** and Trt3 hydroxylases **10** to give **11** (Figure 3). Next, the putative protein Trt6 catalyzes the intra-lactonization reaction to give **12**, while the conversion of **12** to terretonin (**13**) might involve other enzymes that are not identified by Guo et al. (2012).

### Biosynthesis of 10,11-dehydrocurvularin in *A. terreus*

**Figure 4:**
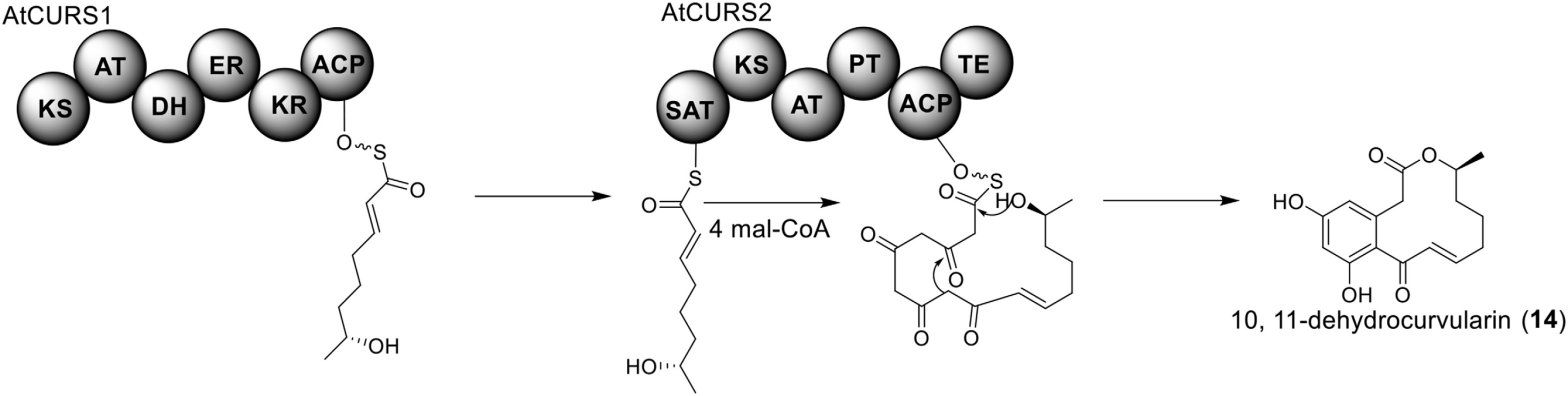
**Biosynthesis of 10,11-dehydrocurvularin in *A. terreus***.

The polyketide origin of the compound 10,11-dehydrocurvularin (**14**) was established by incorporation of stable isotope labeled precursors (Arai et al., 1989). The biosynthetic cluster for 10,11-dehydrocurvularin was identified in *A. terreus* AH-02-30-F7. The cluster is proposed to contain four genes, an HR-PKS gene (*Atcurs1*), an NR-PKS gene (*Atcurs2*), a GAL4-like transcription regulatory gene (*AtcursR*) and a major facilitator superfamily gene (*AtcursE*) (Figure 1) (Xu et al., 2013b). The biosynthesis of 10,11-dehydrocurvularin in *A. terreus* was characterized by heterologous expressing the HR-PKS and NR-PKS pair in *Saccharomyces cerevisiae* (Xu et al., 2013b). In the pathway, the HR-PKS AtCURS1 is proposed to synthesize the tetraketide 7(*S*)-hydroxyotc-2(*E*)-enoic acid (Figure 4). This tetraketide is then loaded onto the starter unit ACP transacylase domain (SAT) of AtCURS2, followed by four chain extension cycles to release the final product **14**. In the cluster, the gene *AtcursR* is predicted to encode for a fungal transcription regulator and *AtcursE* may code for an exporter involved in the transporting of 10,11-dehydrocurvularin (**14**) (Xu et al., 2013b). Further investigation of the product template (PT) domain of AtCURS2, which catalyzes the ring cyclization and aromatization, revealed that the cyclization mode of this domain could be reshaped from the bacterial folding mode, as shown in the biosynthesis of compound **14**, to the fungal mode by three selected point mutations (Xu et al., 2013c). Such rational control of fungal polyketide ring cyclization should facilitate the engineering of natural products with novel chemical scaffolds.

### Biosynthesis of asperfuranone in *A. terreus*

**Figure 5:**
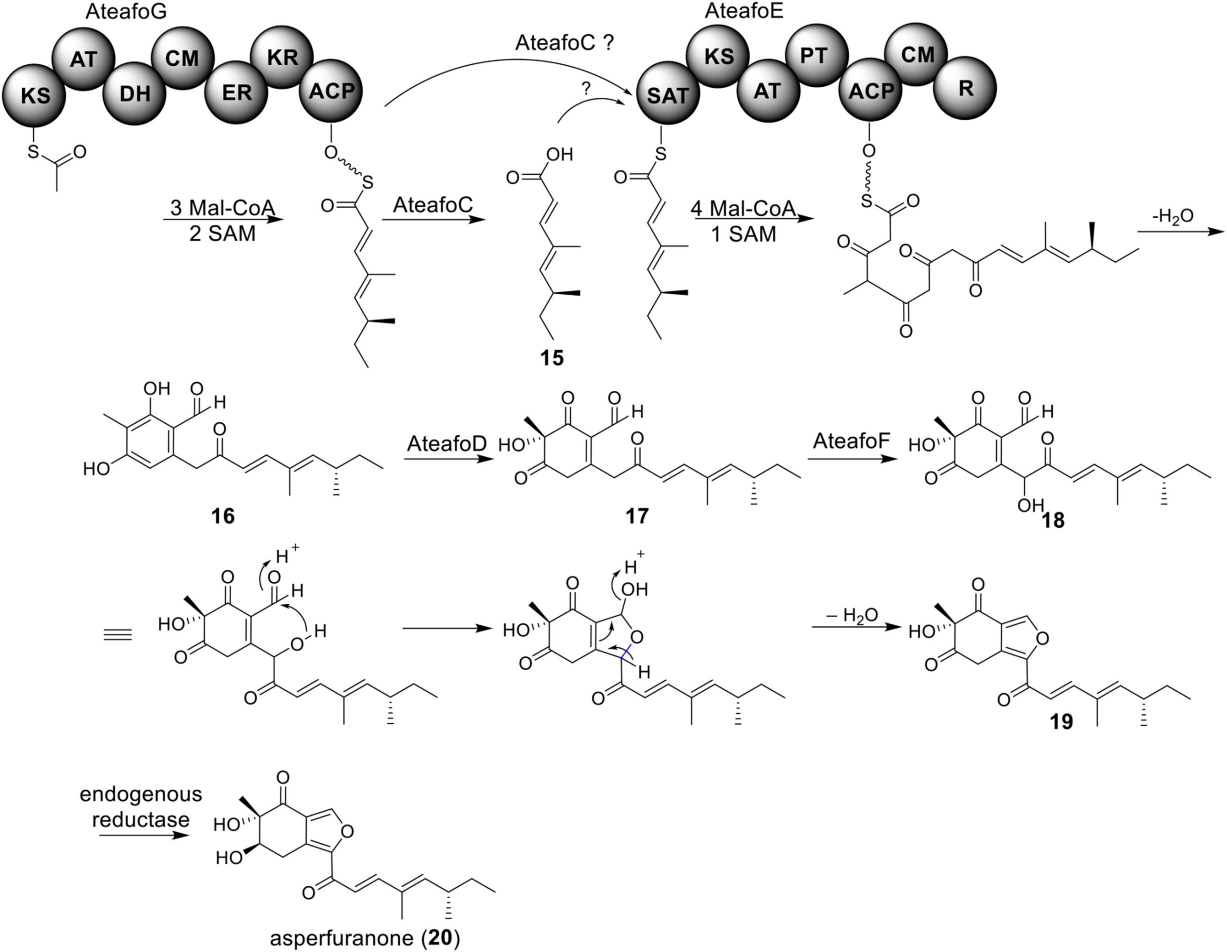
**Biosynthesis of asperfuranone in *A. terreus***.

The azaphilone asperfuranone (**20**) was first isolated from *A. nidulans* after activating two PKSs encoded by *afoE* and *afoG* (Chiang et al., 2009). Later a highly homologous cluster was identified in the *A. terreus* genome (Figure 1) (Chiang et al., 2013). In this case, the individual genes from *A. terreus* were heterologously expressed in *A. nidulans* and the whole asperfuranone pathway was reconstituted. The asperfuranone cluster in *A. terreus* contains a total of seven genes including two PKS genes *ateafoE* and *ateafoG* (Figure 1). In the pathway, the gene *AteafoG* encodes the HR-PKS that is responsible for the production of the PK intermediate **15**. The enzyme AteafoC might facilitate the transfer of the side chain **15** from AteafoG to AteafoE. Next, AteafoD catalyzes the dearomatization of the precursor **16** to give intermediate **17**, followed by hydroxylation by AteafoF to **18**. Last, the authors proposed the existence of an endogenous reductase in *A. nidulans* that catalyzes the conversion of **19** to asperfuranone (**20**) (Figure 5).

### Biosynthesis of terrein in *A. terreus*

**Figure 6:**
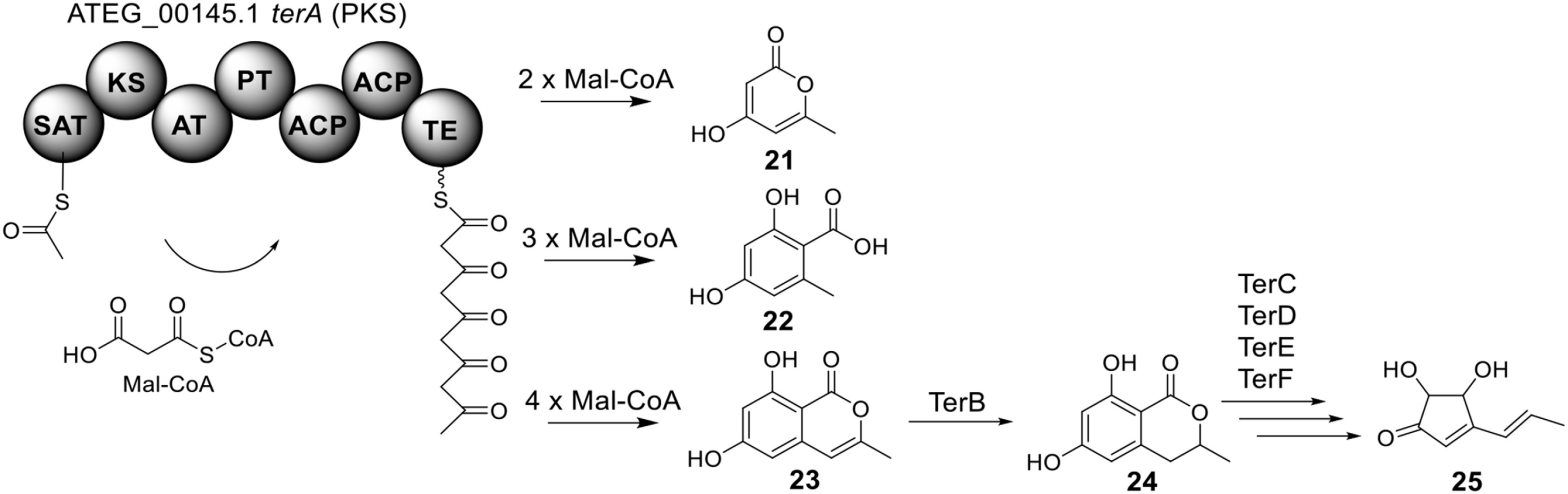
**Biosynthesis of terrein in *A. terreus***.

The terrein (**25**) cluster was serendipitously identified while the Brock group were initially looking for secondary metabolites that are involved in the biosynthesis of conidia pigment in *A. terreus* (Zaehle et al., 2014). Previous isotope-labeling studies revealed that the biosynthesis of terrein originates from a polyketide-based pathway (Birch et al., 1965) and involves the contraction of a six-membered ring precursor to give the five-membered ring terrein (**25**) (Hill et al., 1981). The terrein cluster contains a total of 11 genes, including an NR-PKS gene *terA* (ATEG_00145.1) and a transcription factor gene, *terR* (Figure 1). In the terrein biosynthetic pathway as shown in Figure 6, the NR-PKS encoded by *terA* condensates one acetyl-CoA and four malonyl-CoA to synthesize the precursor 2,3-dehydro-6-hydroxymellein (**23**). Heterologous expression of *terA* in *A. niger* revealed that the NR-PKS TerA is also capable of producing compounds **21** and **22** by incorporating different number of malonyl-CoAs as substrates. The reduction of compound **23** to 6-hydroxymellein (6-HM) (**24**) is probably catalyzed by TerB, but it is also possible that other unspecific ketoreductases may perform this reduction. The authors proposed that the conversion of 6-HM (**24**) to the final product terrein (**25**) might involve four genes, *terC*, *terD*, *terE*, and *terF* while the specific roles of these genes await further verification. Interestingly, the study also revealed that terrein (**25**) has phytotoxic activity. In the two experiments, terrein (**25**), rather than the intermediates or shunt products, inhibited root elongation and caused lesions on fruit surface (Zaehle et al., 2014), representing a prime example of how fungal secondary metabolites might play a role in fungus-plant interactions.

### Biosynthesis of terreic acid in *A. terreus*

**Figure 7:**
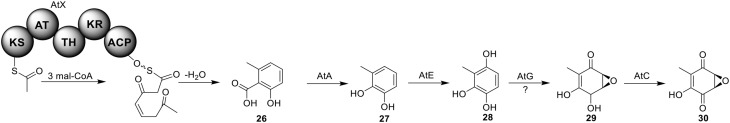
**Biosynthesis of terreic acid in *A. terreus***.

Terreic acid (**30**) is a natural product isolated from *A. terreus* with anti-bacterial activity (Yamamoto et al., 1980). A later study recognized its inhibitory effect against Bruton's tyrosine kinase (Btk), and this compound has been used as a chemical probe to examine the function of Btk (Kawakami et al., 1999). Previous research using isotope-labeled precursors has shown that the biosynthesis of terreic acid originated from one polyketide, 6-methylsalicylic acid (6-MSA, **26**) (Read and Vining, 1968; Read et al., 1969). The PKS gene *atX* was cloned and identified as a 6-MSA (**26**) synthase (Fujii et al., 1996). A recent bioinformatic analysis study predicts that the PKS gene *atX* and its surrounding genes encodes the biosynthetic cluster which is responsible for terreic acid biosynthesis (Boruta and Bizukojc, 2014). The cluster was experimentally characterized using a targeted gene deletion method (Guo et al., 2014). The cluster contains a total of eight genes, including a partially reducing PKS (PRPKS) gene, *atX*, and a transcription factor gene *atF* (Figure 1). The pathway starts with the formation of 6-MSA (**26**) by AtX using one acetyl-CoA and three malonyl-CoA units as its substrates, followed by a decarboxylative hydroxylation catalyzed by AtA to give **27**. Compound **27** could be degraded to a shunt product, (2*Z*,4*E*)-2-methyl-2,4-hexadienedioic acid. The degradation of **27** may be catalyzed by a putative catechol 1,2-dioxygenase in *A. terreus*, but the coding gene for this enzyme is not identified in the terreic acid cluster (Guo et al., 2014). The hydroxylation of **27** to give **28** is possibly catalyzed by AtE. The oxidative conversion of **28** to **29** might involve AtG, but this proposal is not confirmed in the study. Last, AtC catalyzes the oxidation of compound **29** to give the final product terreic acid (**30**) (Figure 7).

### Identification of the products of six NR-PKS genes in *A. terreus*

The previous studies successfully linked several PKS-derived SMs with their biosynthetic clusters in *A. terreus*. Using a heterologous expression strategy, researchers also identified the products of six NR-PKS genes in *A. terreus* (Chiang et al., 2013). The products of these NR-PKSs are shown in Table 1. The NR-PKS encoded by ATEG_00145.1 synthesizes a mixture of compounds, **21**, **22**, **23**, and **31**. The plasticity of ATEG_00145.1 is also demonstrated in the study of terrein biosynthesis (Zaehle et al., 2014). The products of the NR-PKS, encoded by ATEG_03432.1, are compounds **32** and **33**, and the product of the ATEG_03629.1 encoded NR-PKS is **34**. The NR-PKS atrochrysone carboxylic acid synthase (ACAS) encoded by ATEG_08451.1 produces atrochrysone **35** and its derivative emodin **36** with the aid of a β-lactamase encoded by ATEG_08450.1. This result matches the previous finding reported by Awakawa et al. (2009) As expected, ATEG_10080.1 (*trt4*) synthesizes and releases the PKS product 3,5-dimethylorsellinic acid (**4**), which is presumed to be the first precursor in the biosynthesis of terretonin (Figure 3) (Guo et al., 2012; Itoh et al., 2012). As aforementioned, the products of the NR-PKS AteafoE and its adjacent HR-PKS AteafoG incorporate into the asperfuranone pathway as shown in Figure 5 (Table 1).

**Table 1:** **The core synthesis genes and their products identified in *A. terreus***.

**Genes**	**Name**	**Type**	**Putative domain architecture**	**Released product**	**Downstream metabolites**
ATEG_00145.1	*terA*	NR-PKS	SAT-KS-AT-PT-ACP-ACP-TE	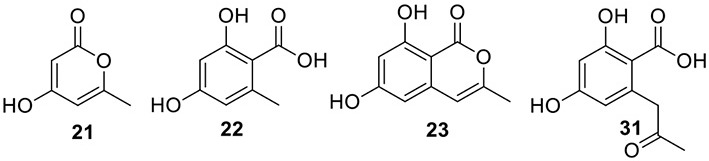	Terrein
ATEG_00228.1		NRPS	Multidomains		
ATEG_00282.1		HR-PKS	KS-AT-DH-KR-ACP-C		
ATEG_00325.1		PKS-NRPS hybrid	KS-AT-(DH)-(CMeT)-(KR)-ACP-C-A-T-R/C	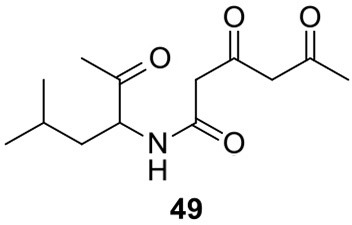	Isoflavipucine
ATEG_00700.1	*atqA*	NRPS-like	A-T-TE	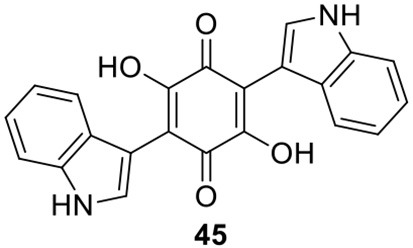	Asterriquinones
ATEG_00881.1		NRPS	A-T-C		
ATEG_00913.1		Undefined	KS-AT		
ATEG_01002.1		NRPS	A-T-C-C-A-T-C-A-T-C-A-T-R		
ATEG_01052.1		NRPS-like	A-T-R		
ATEG_01894.1		HR-PKS	KS-AT-DH-ER-KR-ACP		
ATEG_02004.1	*apvA*	NRPS-like	A-T-TE	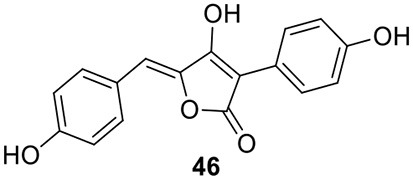	Aspulvinones
ATEG_02403.1		NRPS-like	A-T-R-KR		
ATEG_02434.1		HR-PKS	KS-AT-DH-ACP		
ATEG_02815.1	*btyA*	NRPS-like	A-T-TE	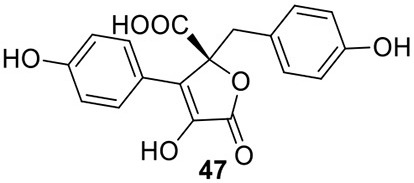	Butyrolactones
ATEG_02831.1		NRPS	A-T-C		
ATEG_02944.1		NRPS	C-A-C-A-T-C		
ATEG_03090.1		NRPS-like	A-T		
ATEG_03432.1		NR-PKS	SAT-KS-AT-PT-ACP-CMeT-R	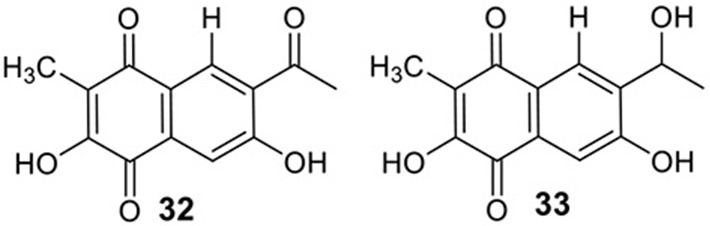	
ATEG_03446.1		HR-PKS	KS-AT-DH-CMeT-ER-KR-ACP		
ATEG_03470.1	*ataP*	NRPS	T-C-A-T-C	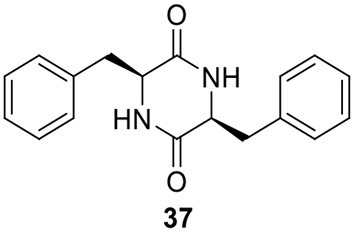	Acetylaranotin
ATEG_03528.1		NRPS	A-T-C-A-T-C		
ATEG_03576.1		NRPS	C-A-T-C-A-T-C		
ATEG_03629.1		NR-PKS	SAT-KS-AT-PT-ACP-ACP-CMeT-TE	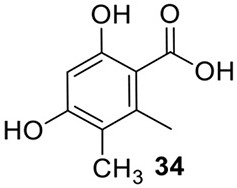	
ATEG_03630.1		NRPS-like	A-T-R	Reduce **34** to its aldehyde form	
ATEG_04322.1		NRPS	C-A-T-C		
ATEG_04323.1		NRPS	A-T-C-C-A		
ATEG_04718.1		HR-PKS	KS-AT-DH-KR-ER-KR-ACP		
ATEG_04975.1		NRPS-like	A-T-R		
ATEG_05073.1		NRPS	A-T-C-A-T-C-T-C-T-C		
ATEG_05795.1		NRPS-lke	A-T-R		
ATEG_06056.1		HR-PKS	KS-AT-DH-CMeT-ER-KR-ACP		
ATEG_06113.1		NRPS	A-T-C-C-A-T-C		
ATEG_06206.1		Undefined	KS-AT		
ATEG_06275.1	*atX*	PRPKS	KS-AT-TH-KR-ACP	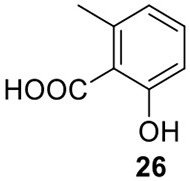	Terreic acid
ATEG_06680.1		HR-PKS	KS-AT-DH-CMeT-ER-KR-ACP		
ATEG_06998.1		NRPS-like	A-T-R		
ATEG_07067.1		HR-PKS	KS-AT-DH-ER-KR-ACP		
ATEG_07279.1		HR-PKS	KS-AT-DH-CMeT-ER-KR-ACP		
ATEG_07282.1		HR-PKS	KS-AT-DH-CMeT-KR-ER-KR-ACP		
ATEG_07358.1		NRPS	A-T-C-A-T-C-T		
ATEG_07379.1		HR-PKS	KS-AT-DH-ACP-ACP-TE		
ATEG_07380.1		NRPS-like	A-T-R		
ATEG_07488.1		NRPS	A-T-C-A-T-C		
ATEG_07500.1		HR-PKS	KS-AT-DH-ACP-ACP-TE		
ATEG_07659.1	*AteafoG*	HR-PKS	KS-AT-DH-CMeT-ER-KR-ACP	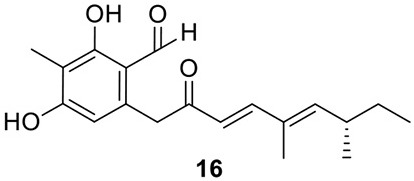	Asperfuranone
ATEG_07661.1	*AteafoE*	NR-PKS	SAT-KS-AT-PT-ACP-CMeT-R		Asperfuranone
ATEG_07894.1		NRPS-like	A-T-R		
ATEG_08172.1		HR-PKS	KS-AT-DH-CMeT-ER-KR-ACP		
ATEG_08427.1		NRPS	A-C-A-T-C-T		
ATEG_08451.1		NR-PKS	SAT-KS-AT-PT-ACP	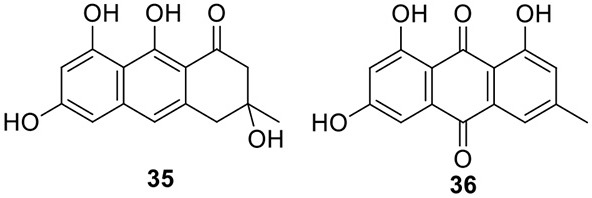	
ATEG_08662.1		NR-PKS	SAT-KS-AT-ACP-CMeT-R		
ATEG_08678.1		NRPS-like	A-T-R		
ATEG_08899.1		NRPS-like	A-T-TE		
ATEG_09019.1		NRPS	C-A-T-C-C-T-A-T-C-A-T-C-A-T-C		
ATEG_09033.1		NRPS-like	A-T-R		
ATEG_09064.1		NRPS	A-T-C-A-T-C		
ATEG_09068.1		NRPS	C-A-T-C-A-T-R		
ATEG_09088.1		HR-PKS	KS-AT-DH-CMeT-ER-KR		
ATEG_09100.1		HR-PKS	KS-AT-DH-CMeT-ER-KR-R		
ATEG_09142.1		NRPS-like	A-T-R		
ATEG_09617.1		HR-PKS	KS-AT-DH-CMeT-KR-ACP		
ATEG_09961.1	*lovB*	HR-PKS	KS-AT-DH-CMeT-(ER)-KR-ACP-CON	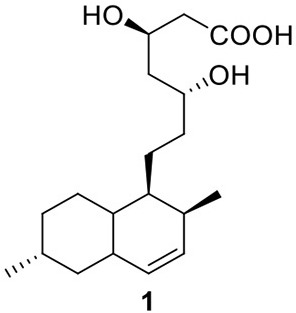	lovastatin
ATEG_09968.1	*lovF*	HR-PKS	KS-AT-DH-CMeT-ER-KR-ACP	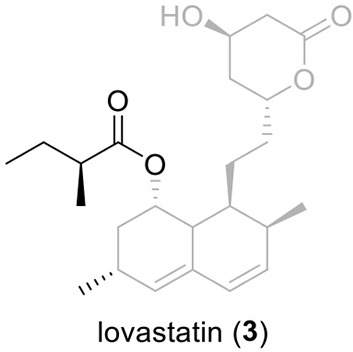	lovastatin
ATEG_10080.1	*trt4*	NR-PKS	SAT-KS-AT-PT-ACP-CMeT-TE	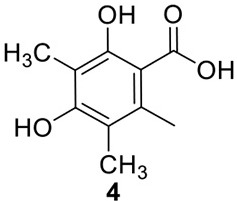	Terretonin
ATEG_10305.1	*anaPS*	NRPS	A-T-C-A-T-C	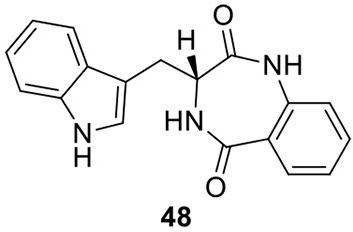	Epi-aszonalenins

*Domains shown in bracket are supposed to be non-functional*.

## Biosynthesis of NRPS-derived natural products in *A. terreus*

### Biosynthesis of acetylaranotin in *A. terreus*

Acetylaranotin belongs to one group of SM toxins named epipolythiodioxopiperizine (ETP). This type of natural products is usually featured by the presence of unique di- or poly-sulfide bridges. The di- or poly-sulfide bridges are presumed to mediate the molecule's cytotoxicity, either by cross-linking vital proteins via cysteine bonds, or by generating reactive oxygen species via redox cycling (Scharf et al., 2011a). An early study using stable isotope-labeled precursors showed that the diketopiperazine (DKP) precursor, compound **37**, could be incorporated intact into acetylaranotin (**44**) (Boente et al., 1981). This indicates that the acetylaranotin pathway may include an NRPS that catalyzes the condensation of two L-phenylalanines to give **37**. Bioinformatic analysis of the ETP clusters dispersed in filamentous ascomycetes uncovered two putative clusters in *A. terreus* that might encode the biosynthetic pathway for acetylaranotin (Patron et al., 2007).

**Figure 8:**
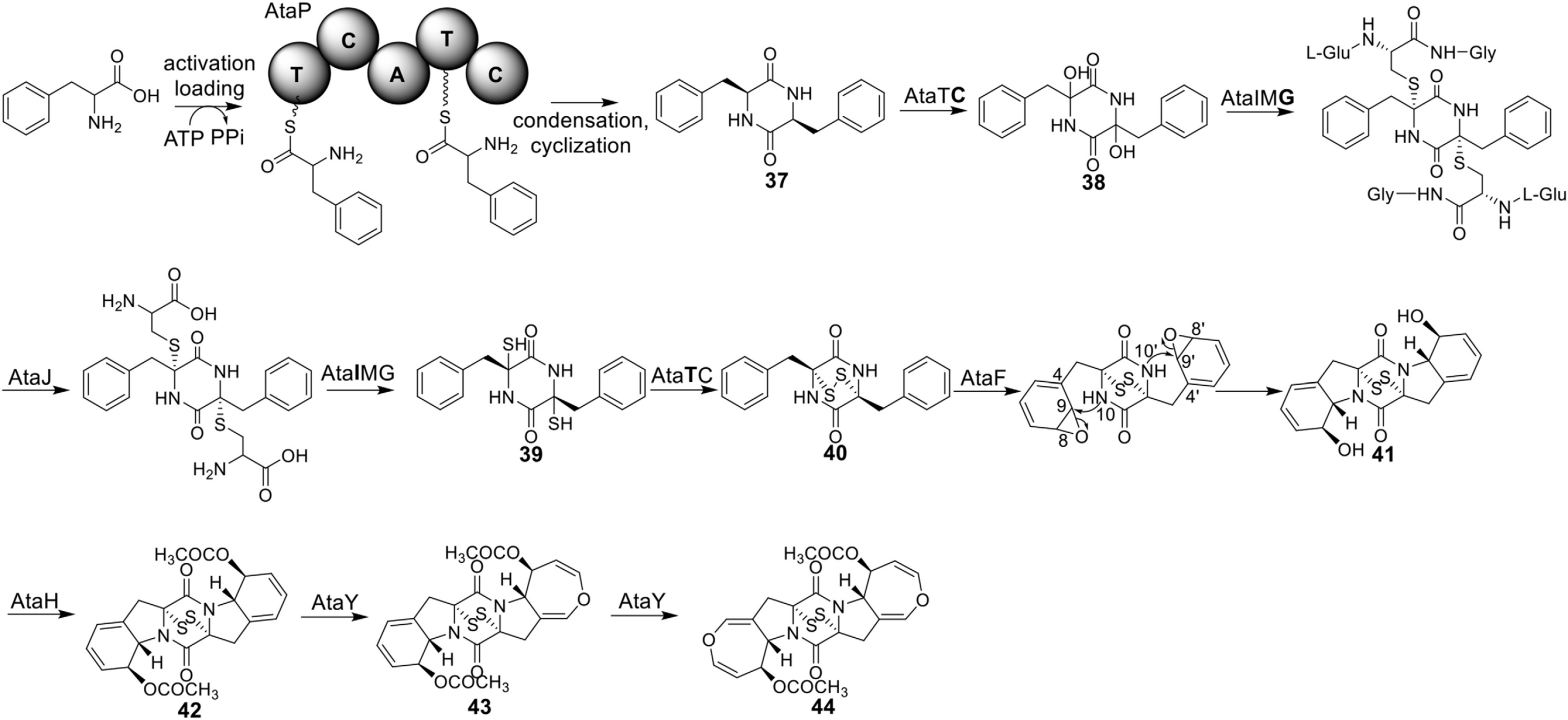
**Biosynthesis of acetylaranotin in *A. terreus***.

Using the gene targeted deletion strategy, one of the two clusters, named *ata* cluster, was found to be responsible for acetylaranotin production in *A. terreus* (Guo et al., 2013b). The *ata* cluster contains a total of nine genes, including one NRPS gene, *ataP* (Figure 1). In the pathway, the NRPS AtaP catalyzes the condensation of two phenylalanines to give compound **37**. Next, the C domain of the protein AtaTC is proposed to catalyze the dual hydroxylation of compound **37** to **38**. A series of studies have fully elucidated the functions of the enzymes that are involved in the installation of the disulfide moiety in gliotoxin (Scharf et al., 2010, 2011b, 2012, 2013; Schrettl et al., 2010; Davis et al., 2011; Gallagher et al., 2012). Similar to their homologs in the gliotoxin cluster, AtaG, AtaJ, AtaI are involved in the conversion of the dual hydroxyl groups in **38** to thiol groups in **39**, followed by the AtaT-mediated oxidation to form the transannular disulfide bridge as shown in intermediate **40**. Next, AtaF catalyzes the dual epoxidation of **40** followed by the spontaneous nucleophilic attack of the amide nitrogens to yield intermediate **41**. The acetylation of **41** to **42** is catalyzed by AtaH, and the oxepine ring formation involves the protein AtaY to yield acetylaranotin **44** (Figure 8). The proteins AtaH and AtaY can function independently, but the detailed catalytic mechanism of AtaY still awaits further investigation. Two recent studies unveiled that the *S*-alkylation of gliotoxin was catalyzed by a SAM dependent methyltransferase encoded by a gene *tmtA/gtmA* outside the gliotoxin cluster (Dolan et al., 2014; Scharf et al., 2014). These studies suggest that the *S*-methylation of acetylaranotin to give bisdethiobis(methylthio)acetylaranotin is likely to be catalyzed by a methyltransferase encoded by a gene outside the acetylaranotin cluster.

### Identification of the products of four NRPS or NRPS-like genes in *A. terreus*

Screening of the genome-sequenced strain *A. terreus* NIH 2624 showed that this fungus is able to biosynthesize four main types of secondary metabolites including aspulvinones, butyrolactones, asterriquinones, and aszonalenins (Guo et al., 2013a). The NRPS-origin of these four types of natural products was revealed in previous studies (Kiriyama et al., 1977; Nitta et al., 1983; Balibar et al., 2007; Yin et al., 2009). To link these molecules to their core synthesis NRPS genes, a mutant library was created by individually deleting a total of 21 NRPS or NRPS-like genes in *A. terreus*. Screening of the mutants' SM profiles, in comparison with that of the wild type, showed that three NRPS-like genes *atqA* (ATEG_00700.1), *apvA* (ATEG_02004.1), and *btyA* (ATEG_02815.1), are responsible for the synthesis of the core structures of asterriquinones, aspulvinones, and butyrolactones, respectively (Table 1) (Balibar et al., 2007; Guo et al., 2013a). The NRPS gene *anaPS* encoded by ATEG_10305.1 is proposed to biosynthesize the (R)-benzodiazepinedione core (Table 1) (Yin et al., 2009; Guo et al., 2013a). Serendipitously, deletion of one NRPS-like gene *atmelA* (ATEG_03563.1) generates an albino mutant that abolishes the brown conidia melanin in *A. terreus*. It is possible that AtmelA synthesizes the first precursor that incorporates into the conidia pigment produced by *A. terreus*. Since all the identified conidial melanins in other *Aspergillus* species are derived from a PKS pathway, another possibility is that this NRPS-like gene might be involved, but not directly related in the melanin biosynthesis of *A. terreus* (Guo et al., 2013a).

### An NRPS-like gene is involved in activating and reducing of an aryl acid released from an NR-PKS

Zhao group recently reported that an NRPS-like protein encoded by ATEG_03630.1 could catalyze the reduction of compound **34** to an aryl aldehyde (Wang et al., 2014). Compound **34** is released by its adjacent NR-PKS ATEG_03629.1, The putative domain architecture of the NRPS-like protein is A-T-R. The aryl acid product **34**, upon its release from the TE domain of the NRPKS, is then loaded onto the A domain. And the R domain is responsible for the reduction of the aryl acid **34** to its aldehyde form (Wang et al., 2014). Interestingly, a subsequent study revealed that the substrate of the A domain of this NRPS-like enzyme could be engineered to anthranilate via bioinformatic analysis and mutagenesis (Wang and Zhao, 2014).

## Biosynthesis of hybrid PKS-NRPS natural products in *A. terreus*

### Biosynthesis of isoflavipucine in *A. terreus*

**Figure 9:**
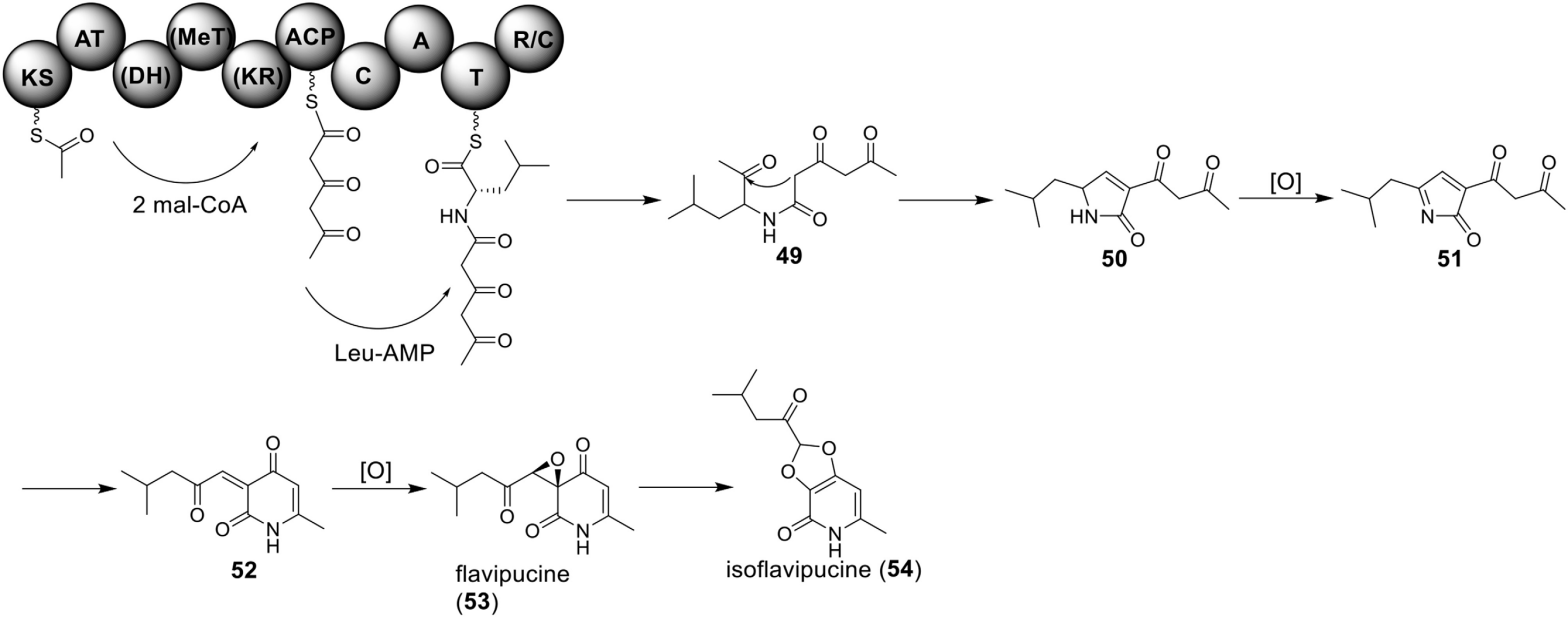
**Biosynthesis of flavipucine in *A. terreus***.

Only one hybrid PKS-NRPS gene, ATEG_00325.1, can be identified in the genome of *A. terreus* NIH 2624. The Brock group identified that the PKS-NRPS encoded by this hybrid gene is responsible for the core synthesis of isoflavipucine and dihydroisoflavipucine (Gressler et al., 2011). RT–PCR analysis of expression of the hybrid gene and its surrounding genes, under isoflavipucine producing condition, suggested that the cluster for isoflavipucine biosynthesis might contain five genes, ATEG_00325.1, ATEG_00326.1 (transcription factor gene), ATEG_00329.1, ATEG_00330.1, and ATEG_00331.1 (Figure 1) (Gressler et al., 2011). In the pathway, the hybrid PKS-NRS synthesizes and releases the linear precursor (**49**). The conversion of the precursor **49** to final product includes heterocyclization (**49** to **50**), oxidation (**50** to **51**), transamination and ring arrangement (**51** to **52**), and epoxidation to give flavipucine (**53**) (Figure 9). However, the enzymes that are responsible for the conversion of the linear precursor **45** to isoflavipucine (**54**) are not identified in this study and needs further investigation.

## Conclusion

In the fungus *A. terreus*, most of the genome mining experiments are carried out in the strain NIH 2624 because it is the only *A. terreus* strain in which the genome sequence is publicly available. A combination of bioinformatic analysis and experimental verification have enabled researchers to elucidate the functions of genes and proteins that are involved in the biosynthesis of several SMs produced in *A. terreus*. However, as shown in Table 1, there are still many other SM genes or gene clusters in *A. terreus* whose products still remain elusive. With the advance of genome sequencing and manipulation techniques, an increasing amount of effort will focus on deciphering the products of these silent or cryptic genes or gene clusters, as well as engineering the characterized pathways to produce second generation molecules with improved bioactivities.

### Conflict of interest statement

The authors declare that the research was conducted in the absence of any commercial or financial relationships that could be construed as a potential conflict of interest.
